# Significance of Abnormal Myocardial Perfusion Scans in Candidates for Orthotopic Liver Transplantation

**Published:** 2017-01

**Authors:** Atefe Esmaili, Saeed Farzanefar, Alireza Emami-Ardakani, Mehrshad Abbasi

**Affiliations:** 1 *Research Institute for Nuclear Medicine, Shariati Hospital, Tehran University of Medical Sciences, Tehran, Iran.*; 2 *Department of Nuclear Medicine, Vali-e-Asr Hospital, Tehran University of Medical Sciences, Tehran, Iran.*

**Keywords:** *Tomography, emission-computed, single-photon*, *Liver transplantation*, *Risk assessment*

## Abstract

**Background:** The implications of cardiac risk stratification before orthotopic liver transplantation (OLT) are not well established. We studied the usefulness of myocardial perfusion imaging (MPI) in this scenario.

**Methods:** MPI data of 24 patients (9 females), candidates of OLT, were collected. They underwent MPI as part of their preoperative risk assessment. MPIs were interpreted by 2 nuclear physicians, who had access to clinical data, scan, and semi-quantification results (i.e., quantitative perfusion single-photon emission tomography (SPECT) [QPS] and quantitative gated SPECT [QGS]). A 3rd nuclear physician, blinded to the clinical history of the subjects, re-reviewed the scans. The visual interpretations of MPI (i.e., normal vs. abnormal), ejection fraction, and transient ischemic dilation index derived from QPS and clinical and follow-up data were collected and analyzed.

**Results:** The follow-up period was 231.0 ± 86.0 days. The MPIs were normal in 16 (66.7%) patients and abnormal in 8 (i.e., 5 mild [20.8%], 1 [4.2%] moderate, and 2 [8.3%] severe). Out of 4 patients who died during the follow-up, 1 had mild ischemia and 2 had severe ischemia. A patient who had a normal MPI died due to noncardiac reasons. A patient with abnormal MPI had 3-vessel disease on angiography. Out of the 5 patients who died or had significant coronary angiographic abnormalities, 4 had abnormal MPIs (negative predictive value = 93.8%; sensitivity = 80.0%) The MPIs of 4 patients without perioperative mortality or cardiac morbidity were abnormal (specificity = 78.9%).

**Conclusion: **MPI seems to be remarkable in discriminating high-risk OLT patients preoperatively.

## Introduction

There is controversial published information regarding the significance of cardiac risk assessment prior to liver transplantation.^1^ Considering the high prevalence of metabolic derangements^2^ in orthotopic liver transplantation (OLT) candidates, a thoughtful cardiac work-up is seemingly essential.^3^ Some scholars perform coronary angiography in the majority of the patients^4^ and others preclude any need for preoperative cardiac risk assessment.^5^ Myocardial perfusion imaging (MPI) has been used for the purpose of the preoperative risk stratification of moderate to high risk surgeries for years.^6^ Nevertheless, there is evidence against the usefulness of MPI for the preoperative cardiac risk assessment of candidates for liver transplantation.^7^ In recent years, liver transplantation has been practiced in Iran in a few centers in Tehran and Shiraz,^8^^, ^^9^ albeit no generally accepted cardiac assessment algorithm exists. We here report the association between MPI results and perioperative mortality of Iranian patients candidated for OLT and discuss the usefulness of MPI in these patients. 

## Methods

The study included 24 patients (9 females; 37.5%) candidated for OLT in a referral teaching university hospital (Tehran, Iran). All the subjects were referred to the Department of Nuclear Medicine for myocardial perfusion scintigraphy between March and September 2012. The indication for MPI was risk stratification before a high-risk surgery. Based on the appropriate use criteria,^10^ the appropriateness of MPI is reasonable in patients with a poor function class and uncertain in those with a good function class and interpretable electrocardiogram. Also, MPI could be informative in the long-term risk assessment and corresponding treatments of the candidates before undergoing expensive and demanding surgeries.^11^ The patients underwent myocardial perfusion gated single-photon emission tomography (SPECT) imaging with a 99m technetium sestamibi tracer and a dual-headed Gama camera (ADAC Forte, USA). In a 2-day stress and rest protocol, about 0.5 mgr/kg/4 minutes Dipyridamole was infused for the stress phase. The MPIs were interpreted by two nuclear physicians, not blinded to the patient history and reason for referral. The quantitative perfusion and gated data were analyzed in Cedars-Sinai Auto QUANT software (LA, California, USA). The survived patients were followed up prospectively for 231 ± 86 (range = 131-451) days, and the perioperative mortality and cardiac events were recorded. The hard copies of the MPIs were then reviewed by a third nuclear physician, who was blinded to the topic of study and the history and cardiac risk profile of the patients. 

The protocol of the study conformed to the ethical guidelines of the 1975 Helsinki Declaration. The data of human subjects were anonymously handled, and no intervention or change was done in patient management for the purposes of the study. For the statistical analyses, the statistical software SPSS version 17.0 for Windows (SPSS Inc., Chicago, IL) was used. The qualitative data are presented as percentages, and the quantitative data are reported as standard deviation (SD). The independent sample t-test and the chi-squared test were used respectively for the analysis of the quantitative and qualitative data between the subjects with and without MPI abnormality. 

## Results

The data of 24 patients candidated for OLT (aged 53.7 ± 9.1 years) were analyzed for this report. Table 1 summarizes the health characteristics of the study participants. The patients had moderate risk of perioperative fatal and nonfatal cardiac events based on the Goldman risk index.^12^ The most common etiology of liver cirrhosis was viral hepatitis (i.e., hepatitis B, C, or D viruses) in 11 subjects. The most common cardiac risk factor was smoking in 7 (29.2%) subjects. MPI was reported normal in 16 (66.7%) patients and abnormal in 8 patients, including 5 cases of mild (20.8%), 1 case of moderate (4.2%), and 2 cases of (8.3%) severe ischemia in at least one territory. The sum stress score of the MPIs with abnormalities was higher than that with normal readings (2.8 ± 2.0 vs. 0.6 ± 0.6; p value = 0.012). The ejection fraction and the index of the transient ischemic dilation produced by the Auto QUANT software were the same in the patients with and without MPI abnormalities (data not shown). Out of 7 smokers (6 males), 3 male subjects had abnormal MPIs. All the abnormal MPIs were reported in the subjects with no history of diabetes, hypertension, and dyslipidemia. Out of 8 subjects with abnormal readings, only one patient was symptomatic (i.e., dyspnea). During the follow-up, 4 patients deceased (2 before surgery); 1 of them had mild ischemia and 2 had severe ischemia. One of those with severe ischemia was not operated on and died while on the waiting list for surgery. The other patient who died before transplantation had a normal MPI and died due to noncardiac reasons. The only patient who underwent angiography (three-vessel disease) had an abnormal MPI and was forwarded for cardiac revascularization before OLT. The review of the MPI by a third nuclear physician, blinded to the history and pretest risk profile of the patients, categorized the MPIs as normal in 17 and abnormal in 7 cases (at least ischemia of one territory; 5 mild and 2 moderate cases). His interpretation (i.e. normal/abnormal) was similar to the reading of the initial interpreters, who had full accesses to the patient history in 21 subjects (87.5%; 2 abnormal scans were interpreted normal and 1 normal scan was interpreted abnormal). Out of 5 patients who died perioperatively or had significant coronary angiographic abnormalities, 4 subjects had abnormal MPIs (negative predictive value = 93.8%; sensitivity = 80.0%); however, the MPIs of 4 patients without perioperative mortality or cardiac morbidity were reported abnormal (specificity = 78.9%; accuracy = 79.2%). The accuracy of the blinded interpretations was 83.3% (sensitivity = 80.0%; specificity = 84.2%; and negative predictive value = 94.1%). All the 5 patients who died perioperatively or had significant coronary angiography were male, and the accuracy of MPI to detect these males was 80.0% (sensitivity = 80.0%; specificity = 80.0%; and negative predictive value = 88.9%).

**Table 1:** Patients’ characteristics*

Sex	
Females	9 (37.5)
Males	16 (62.5)
Age (y)	63.6±9.1
Main cardiac symptoms	
Typical angina	2 (8.3)
Dyspnea	3 (12.5)
No complaint	19 (79.2)
Smoking	7 (29.2)
Dyslipidemia	2 (8.3)
Diabetes	3 (12.5)
Hypertension	1 (4.2)
Cirrhosis etiology	
Autoimmune	1 (4.2)
HBV	6 (25.5)
HBV& HDV	2 (8.3)
HCV	3 (12.5)
Alcoholic	1 (4.2)
NASH	2 (8.3)
Primary biliary cirrhosis	2 (8.3)
Primary sclerosing cholangitis	2 (8.3)
Ryptogenic	3 (12.5)
Myocardial perfusion scan	
Normal	16 (66.7)
Abnormal	
Mild	5 (20.8)
Moderate	1 (4.2)
Severe	2 (8.3)

HBV, Hepatitis B virus; HDV, Hepatitis D virus; HCV, Hepatitis C virus; NASH, Nonalcoholic steatohepatitis

* Data are presented as mean±SD or n (%).

## Discussion

The necessity and the method of choice for preoperative cardiac evaluation in OLT candidates are not yet well determined. The American Association for the Study of Liver Diseases in 2005 suggested that patients with hemochromatosis undergo cardiac evaluation. Dobutamine stress echocardiography would be the preferable method, and catheterization should be performed in those with abnormal stress tests.^13^ Nevertheless cardiovascular disease is a significant cause of death in OLT recipients, and long-term cardiac events occur in 25-70% of subjects.^14^ Hence, preoperative cardiac risk evaluation is reasonably required while the preferable method is debatable. MPI is more sensitive than Dobutamine echocardiography for detecting ischemia in patients with liver disease.^15^ In the present study, we demonstrated high sensitivity (80.0%) and specificity (78.9%) for the preoperative risk stratification of candidates for OLT with MPI. This figure is fairly similar to the risk assessment profile of the MPI in the general population. Our result supports the reports suggesting the value of MPI for the preoperative risk assessment of OLT candidates^16^^-^^18^ and is in contrast to those findings indicating that screening MPI results do not affect operation eligibility^19^ or are limited.^5^ Our results are essentially applicable in male patients. 

The prevalence of perioperative hard cardiac events or cardiac-related mortality for OLT candidates is reported to be between 3 and 23%.^20^ In our study, 4 patients died and one patient had abnormal angiography accounting for 20.8% of our patients. We reported 33.3% of the MPIs abnormal (29.2% abnormal reports by the blinded reader). The rate of abnormal reports of MPI in OLT candidates varies widely between studies and is reported form 8%^21^ to 68.8%.^14^ It depends on the sensitivity and specificity of the criteria used and the consistency of the interpretations of the noticed myocardial uptake abnormalities. We verified the consistency of our interpretations with the interpretations of a third nuclear physician, blinded to the history and complaints of the patients, who reported the MPIs very similar to the prior readings (87.5% similarity for abnormal/normal interpretations). The relatively high accuracy of our MPIs to predict perioperative mortality of OLT candidates also verifies the proper sensitivity and specificity of the method. 

The prevalence rates of diabetes and smoking in our OLT candidates were similar to the nationwide prevalence rates of these risk factors (correspondingly 8.7 and 12.5 for diabetes, smoking, and hypercholesterolemia; Iran adult population [2007]).^22^^, ^^23^ Nevertheless, the prevalence rates of hypertension and dyslipidemia were considerably lower than those in the general Iranian population (i.e. correspondingly 25% and 43 % for hypertension and hypercholesterolemia; adult Iran population [2007]).^24^^, ^^25^

Our study suffers from remarkable drawbacks, first and foremost among which is its small sample size. Another limitation is the low frequency of catheterization. Moreover, certain information, including the medication of the patients, should have been recorded during the follow-up period. Finally, the short follow-up in our study is also a considerable weakness. Even so, we can conclude that MPI is an accurate method for the predication of perioperative mortality in OLT candidates. 

## Conclusion

To sum up, we suggest that the prevalence of abnormal myocardial perfusion findings is relatively high and the abnormalities may correlate with the prognosis of the patients. Accordingly, the test should be incorporated into the pre orthotropic liver transplantation risk assessment workup. 

## Notes:

***This paper should be cited as:*** Esmaili A, Farzanefar S, Emami-Ardakani A, Abbasi M. Significance of Abnormal Myocardial Perfusion Scans in Candidates for Orthotopic Liver Transplantation. J Teh Univ Heart Ctr 2017;12(1):23-26.
